# Investigation of key signaling pathways and appropriate diagnostic biomarkers selection between non-invasive to invasive stages in pancreatic cancer: a computational observation

**DOI:** 10.25122/jml-2022-0067

**Published:** 2022-09

**Authors:** Hamid Taghvaei Javanshir, Mohammad Amin Malekraeisi, Seyedeh Sanaz Seyed Ebrahimi, Ahmad Bereimipour, Sara Fakharian Kashani, Amir Abbas Bostaki, Habibollah Mahmoodzadeh, Karim Nayernia

**Affiliations:** 1 Cancer Research Center, Tehran University of Medical Sciences, Tehran, Iran; 2 Department of Biology, School of Basic Science, Science and Research Branch, Islamic Azad University, Tehran, Iran; 3 Student Research Committee, School of Medicine, Iran University of Medical Sciences, Tehran, Iran; 4 Kurdistan Immunology & Haematology Research Center, Kurdistan University of Medical Sciences, Sanandaj, Iran; 5 Department of Stem Cells and Developmental Biology, Cell Science Research Center, Royan Institute for Stem Cell Biology and Technology, ACECR, Tehran, Iran; 6 Medical Genomics Research Center, Tehran Medical Sciences Islamic Azad University, Tehran, Iran; 7 International Center for Personalized Medicine, Düsseldorf, Germany

**Keywords:** biomarkers discovery, non-invasive, invasive, pancreatic cancer, bioinformatics analysis, ECM

## Abstract

Pancreatic cancer is the seventh most lethal cancer in the world. Despite its moderate prevalence, the 5-year survival rate of patients with pancreatic cancer is about 10%. Despite different therapeutic and diagnostic strategies for pancreatic cancer, this cancer is still uncontrollable in the invasive stage and can invade various body organs and cause death. Early detection for pancreatic cancer can be an excellent solution to manage treatment better and increase patients' survival rates. This study aimed to find diagnostic biomarkers between non-invasive to invasive stages of pancreatic cancer in the extracellular matrix to facilitate the early diagnosis of this cancer. Using bioinformatics analysis, we selected the appropriate datasets between non-invasive and invasive pancreatic cancer stages and categorized their genes. Then, we charted and confirmed the signaling pathways, gene ontology, protein relationships, and protein expression levels in the human samples using bioinformatics databases. Cell adhesion and hypoxia signaling pathways were observed in up-regulated genes, different phases of the cell cycle, and metabolic signaling pathways with down-regulated genes between non-invasive and invasive pancreatic cancer stages. For proper diagnostic biomarkers selection, the overexpressed genes that released protein into the extracellular matrix were examined in more detail, with 62 proteins selected and SPARC, THBS2, COL11A1, COL1A1, COL1A2, COL3A1, SERPINH1, PLAU proteins chosen. Bioinformatics analysis can more accurately assess the relationship between molecular mechanisms and key actors in pancreatic cancer invasion and metastasis to facilitate early detection and improve treatment management for patients with pancreatic cancer.

## INTRODUCTION

Pancreatic cancer is one of the most dangerous cancers in the world. Notwithstanding the fair prevalence of this cancer, it has high mortality in the invasive stage and is generally considered the seventh cancer worldwide [1]. The incidence and mortality of pancreatic cancer correlated with increasing age were slightly more common in men than women [2]. In the early stage of pancreatic cancer, symptoms usually remain hidden. Even during the tumor's progression, it only expresses non-specific symptoms such as jaundice, weight loss, light-colored stools, abdominal pain, and fatigue. In addition, the available diagnostic tests are not specific and accurate for the early detection of pancreatic cancer [2–4]. Different approaches against pancreatic cancer include surgery, chemotherapy, and radiotherapy. However, for invasive stage cancer (metastasis phase) patients, the survival chance is meager (5 years survival: about 10%) [5]. Because of the challenges of early diagnosis and its importance, more efficient and higher accuracy methods are still required.

Standard methods for pancreatic cancer, including abdominal ultrasonography, tri-phasic pancreatic-protocol computerized tomography) CT) [5, 6], magnetic resonance imaging (MRI) [3, 7, 8], and endoscopic ultrasound-guided fine-needle aspiration, are typical for patients [3]. These techniques have not been routinely used in the community for the early detection of pancreatic cancer and cannot be accurate [9]. Over the past decade, our increased knowledge about tumor biology cleared new horizons in front of scientists to develop novel and more effective therapeutic [10–12] and diagnostic methods [8, 13–15] for cancer. In this regard, these modern methods for the early detection of cancers, particularly pancreatic cancers, can help manage treatment and increase patients' survival. The discovery of biomarkers has been one of the ideal clarifications for cancer in recent years, widely welcomed by researchers and physicians [16–18].

Nowadays, bioinformatics tools are one of the introductory and powerful applications for studying complex cancer biology [19]. Primarily, these applications can be used to find specific biomarkers, being already used in some studies [18, 20]. This study's primary goal was to detect potential extracellular matrix (ECM) biomarkers in primary pancreatic cancer to diagnose the disease early. The 8 final genes that are indicated through bioinformatics analysis have a great chance of becoming useful in the early diagnosis of pancreatic cancer.

## MATERIAL AND METHODS

### Gene datasets

Microarray datasets for human species were extracted from GEO (www.ncbi.nlm.nih.gov/geo/). Two independent datasets, GSE62165 and GSE19281, were selected. The samples from the datasets were divided into three groups: control, primary, and metastatic stages. In GSE62165 with platform GPL13367 [(HG-U219) Affymetrix Human Genome U219 Array], stage I and stage IV were considered primary and metastatic, respectively. The number of samples was 13 for control and metastatic groups and 8 for primary. The samples of other stages were excluded. GSE19281 had platform GPL96 [(HG-U133A) Affymetrix Human Genome U133A Array], and normal pancreas samples were determined as control. The control group, primary group, and metastatic group had 3, 4, and 5 samples. Datasets had no particular interventions.

### Preparation and clustering of differentially expressed genes [DEGs] for future analysis

Web-based tool GEO2R (www.ncbi.nlm.nih.gov/geo/geo2r/) was used for dataset analysis and identification of DEGs. Each dataset had two comparisons, the first was between control and primary groups, and the second was for control and metastasis. Intersected DEGs of two comparisons were identified using the Venn diagram. The package VennDiagram version 1.6.20 was used for Venn diagrams. Then, common DEGs were considered by the intersection between the two datasets. P-value<0.05 and |logFC|>0 were applied for DEGs selection. Clustering analysis for 200 top-up-regulated genes of each dataset was done by heatmap package version 1.0.12 of R.

### Signaling pathways and gene ontology [GO] enrichment analysis

Enrichr (maayanlab.cloud/Enrichr/), an excellent tool for information analysis of extracted data from different databases, was used to analyze signaling pathways related to up and down-regulated genes. KEGG (www.genome.jp/kegg/) was chsen to predict the signaling pathways associated with DEGs. GO analysis is an effective method to predict the biological characteristics of selected genes, which includes: biological process (BP), molecular function (MF), and cellular component (CC). For this purpose, up and down-regulated DEGs were uploaded in the Panther database (pantherdb.org/), a classification web system to classify genes and proteins, then GO performed analysis. However, analyzing cellular components was applied just for up-regulated genes. P-value<0.05 was applied for all studies.

### Protein-Protein Interactions [PPIs] analysis

Genes with CC belonging to extracellular matrix entered to STRING database version 11.0 (string-db.org/). The resulting PPIs network was sent to Cytoscape software version 3.8.2 and analyzed topologically using Cytohubba version 0.1. Thirty top genes of 12 algorithms were identified, and hub genes were chosen from the top genes best for 12 algorithms.

### Investigation of hub genes in cancer databases

GEPIA (gepia.cancer-pku.cn/), based on the TCGA database, is an online tool to study and analyze cancer-regulated genes. In this study, the hub genes were considered to examine their roles and performances in pancreatic ductal adenocarcinoma (PAAD). A box plot visualized expression differences between pancreatic and control samples. Furthermore, the Kaplan-Meier method was performed for survival analysis of the hub genes. Based on the results, the genes with the most mortality influence were included in the PAAD report. P<0.05 was considered statistically significant.

## RESULTS

### Evaluation of DEGs between non-invasive and invasive pancreatic cancer

**Figure 1:**
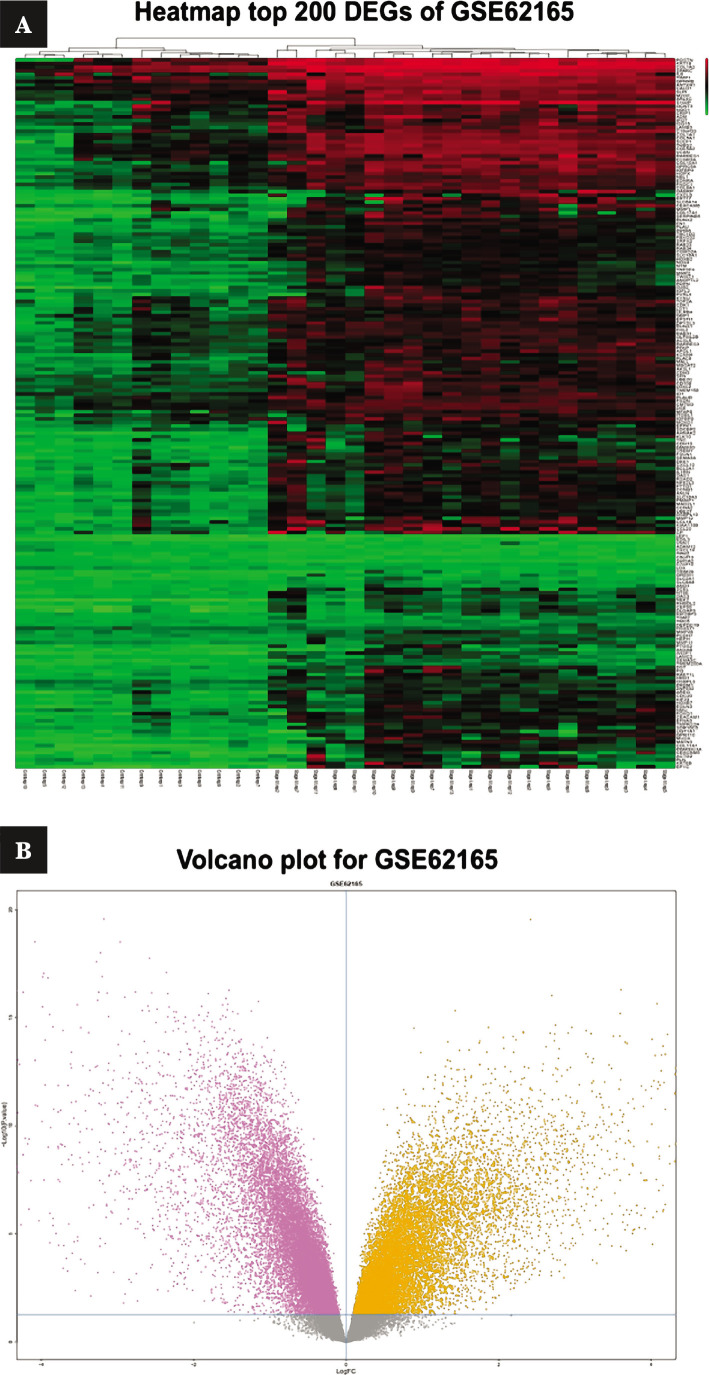
Investigation of gene expression differentiation in selected datasets in which the top 200 genes are plotted as: A – heatmap and B – volcano plot.

Each of the datasets, GSE62156 and GSE19281, had two comparisons. For GSE62165, the first comparison was between control and primary samples, and the second was between control and metastatic samples. The intersection of the two comparisons identified 5887 up-regulated genes and 8487 down-regulated genes. GSE19281 had 1049 and 1377 up-regulated and down-regulated genes, respectively. Venn diagram obtained 1727 common DEGs where 798 genes were up-regulated, and 929 genes were down-regulated. However, primary samples were among the metastatic samples. On the other hand, genes that belonged to the extracellular matrix were chosen from up-regulated genes that show that extracellular matrix genes can differentiate healthy samples from cancer (Figure 1 A, B).

### Signaling pathways of cell adhesion and hypoxia were observed significantly between non-invasive and invasive pancreatic cancer

**Figure 2:**
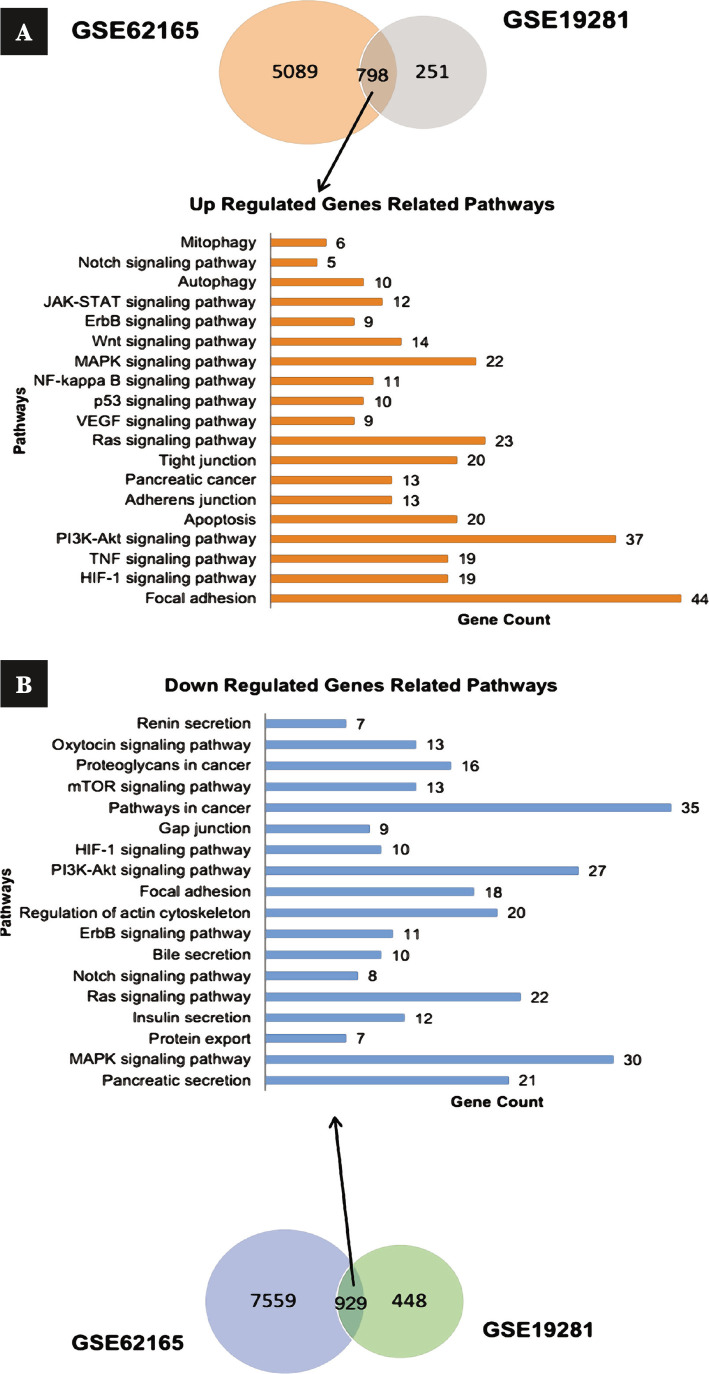
Common genes between datasets are isolated, and the selected signaling pathways of each are shown along with the number of genes involved in the pathways. A – up-regulated genes; B – down-regulated genes.

To investigate the functions of DEGs, KEGG and GO, analyses of up and down-regulated DEGs were conducted. Generally, 19 cancer-regulated pathways with P<0.05 were detected in KEGG, which revealed that the most significant up-regulated pathways were focal adhesion (P=7.76E-21), ECM-receptor interaction (P=6.6E-13), HIF-1 signaling pathway (P=1.34E-08), TNF signaling pathway (P=6.69E-08), PI3K-Akt signaling pathway (P=9.78E-08) and apoptosis (P=1.05E-06). Conversely, down-regulated pathways mainly included pancreatic secretion (P=3.44E-09), MAPK signaling pathway (P=5.08E-05), protein export (P=5.81E-05), insulin secretion (P=0.000604), Ras signaling pathway (P=0.00124), and Notch signaling pathway (P=0.001519) (Figure 2 A, B).

### Gene ontology analysis

**Figure 3:**
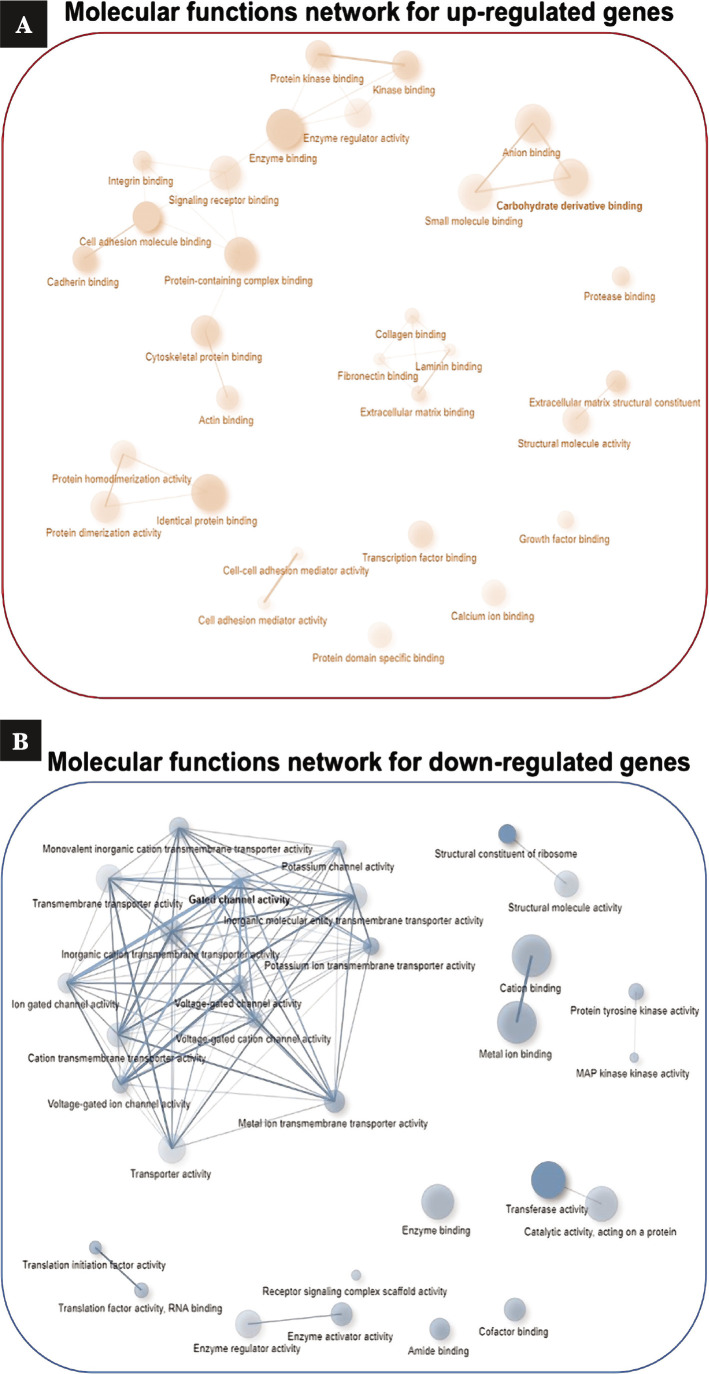
Drawing a network of molecular functions that shows the importance and relationship between genes' activity. Large and bold circles show more significance than lighter and smaller circles. A – up-regulated genes; B – down-regulated genes.

To conduct GO analysis, up and down-regulated DEGs were entered in the Panther database, and then BP and MF analysis was conducted for up and down-regulated DEGs. Still, CC was just performed for up-regulated DEGs. Tables 1 and 2 present that biological processes of up-regulated DEGs primarily included cell migration (FDR=8.53E-39), response to stress (FDR=2.53E-37), cell motility (FDR=2.53E-37), biological adhesion (FDR=4.49E-33), cell differentiation (FDR=5.11E-32), cell surface receptor (FDR=4.50E-30). The most enriched down-regulated DEGs in BP were protein targeting (FDR=1.15E-14), cellular localization (FDR=9.96E-11), regulation of transport (FDR=3.54E-10), cellular protein localization (FDR=4.71E-10), cell-cell signaling (FDR=1.86E-08), and cellular catabolic process (FDR=3.09E-08). The binding was the most enriched GO term in MF for both groups, including most gene numbers. CC analysis of up-regulated genes was performed to detect the DEGs with a high ECM expression level in PAAD cancer. Findings revealed that 62 DEGs were related to ECM; therefore, these 62 genes were considered for further analysis (Figure 3 A, B, Tables 1, 2).

**Table 1:** Biological processes in up-regulated genes.

Term	P-value cut off [FDR]	Top 50 Genes
Cell migration	8.53E-39	PLXND1/TMSB10/NDE1/ITGB5/PLAT/MET/NEDD9/VEGFA/PLXNA1/CCL20/SDC1/RND3/TNFAIP3/SDC4/RAC2/ITGB4/ANXA1/RAC1/IL1RN/ARPC5L/LEF1/ITGB1/THY1/AUTS2/CXCL5/PAK2/HMGB1/PLXNB2/TMSB4X/ADAM17/MYLK/CEACAM1/FERMT1/SERPINE1/GATA3/COL1A1/SPARC/FN1/ IGFBP5/LGALS8/PLAU/EFNB2/APOE/LGALS3/KLF4/SORL1/THBS1/ ENPEP/PLA2G7/IGFBP3
Response to stress	2.53E-37	ACTB/MYL12A/ILK/RECQL/SBNO2/TNFRSF1A/MAP4K4/TRAF4/HSP90AA1/PPP1R15A/CDC7/USP10/RIPK2/UBR5/BNIP3L/VEGFA/BCL6/CCL20/PRDX1/PLAU/TNF/AIP6/UBA1/PCNA/ANXA1/OASL/MAP3K7/IL1RN/DUOX2/PML/IFITM3/SOD1/MCL1/MSRB2/HMGA2/DST/TNIK/REL/CXCL5/HMGB2/OTUD4/NIPBL/CD14/FPR1/PRNP/PLK3/PLEC/PAK2/ANXA2/IFITM2/IFITM1
Cell motility	2.53E-37	PLXND1/TMSB10/NDE1/ITGB5/PLAT/MET/NEDD9/VEGFA/PLXNA1/CCL20/SDC1/RND3/TNFAIP3/SDC4/RAC2/ITGB4/ANXA1/RAC1/IL1RN/ARPC5L/LEF1/ITGB1/THY1/AUTS2/CXCL5/PAK2/HMGB1/PLXNB2/TMSB4X/ADAM17/MYLK/FBLN1/CEACAM1/FERMT1/SRPX2/SERPINE1/GATA3/COL1A1/SPARC/FN1/IGFBP5/LGALS8/PLAU/EFNB2/APOE/LGALS3/KLF4/SORL1/THBS1/ENPEP
Biological adhesion	4.49E-33	ACTB/MYL12A/ILK/PLXND1/CDH3/ITGB5/ICAM1/ECM2/CDH6/PLXNA1/TGFBI/PLAU/PALLD/COL5A1/ITGB4/POSTN/IL6ST/ANXA1/LEF1/CDH11/TNFRSF21/ITGB1/THY1/PCDH7/PLXNB2/CD47/SIRPA/SPON1/PPP1R12A/CDC42/TFRC/FBLN1/CEACAM1/FERMT1/SRPX2/CEACAM5/CAV1/SERPINE1/VEGFA/FN1/CD58/EFNB2/CD93/ADAM19/NT5E/RAC1/IL1RN/KLF4/THBS1/EPHA2
Cell differentiation	5.11E-32	VCAN/TNC/PSMC2/FGR/PLXND1/ETV1/GAS7/FYN/MAP4/SBNO2/MAP4K4/WDR1/TCF3/RARB/SSH1/ELF4/ANKRD27/CAV2/CAV1/MET/WNT2/GATA3/ELK3/PLXNA1 RTN4/ID2/SOX4/EFNB2/RAC2/LIF/PALLD/ANXA1/RAC1/SKIL/SULF1/ANXA7/TMOD3/LEF1/ABHD2/COL6A2/EPHA2/EGFR/TCF7L2/THY1/TNIK/ETS2/AUTS2/ELF3/HEY1/ARF6/ILK
Cell surface receptor signaling pathway	4.50E-30	FGR/CFLAR/PLXND1/RHBDF1/FYN/GPRC5A/ITGB5/PLAT/CAV2/MET/WNT2/BMPR1A/COL1A1/VEGFA/CSNK1A1/CBLB/PLXNA1/CCL20/IGFBP5/TSPAN1/TNFAIP3/TNFRSF10B/EFNB2/LIF/ITGB4/CSNK1G2/COL4A2/ANXA1/TSPAN31/SKIL/IL1RN/SULF1/LEF1/IGFBP4/IFITM3/EPHA2/BCL10/MCL1/PIK3R1/EGFR/IGFBP3/TCF7L2/ITGB1/THY1/IFNGR2/SHC1/CXCL5/MST1R/OTUD4
Cellular response to cytokine stimulus	7.12E-30	SBNO2/CCL20/IL1RN/IFITM3/IFNGR2/CXCL5/OTUD4/IFITM2/IFITM1/SP100/HIF1A/NFKBIA/GAPDH/STAT1/CD58/GBP1/IRF1/ACTN4/IL6ST/LEF1/DUOX2/TNFRSF21/GBP2/TFPI/PAFAH1B1/TPR/VAMP3/WNK1/PFKP/TNFRSF1A/CDC42/TFRC/IL4R/ICAM1/KLF5/RIPK2/CAV1/PCOLCE/GATA3/COL1A1/OAS2/ACTR3/KMO/KDM5B/CREB1/TUBA1B/NMI/PXDN/ASS1/IL13RA1/
Regulation of signal transduction	1.54E-29	ATP2C1/TRIM38/ECM1/SLC20A1/CANT1/ZDHHC17/CFLAR/RHBDF1/GPRC5A/TRIB2/TRAF4/RIPK2/CAV2/CSNK1A1/CBLB/CCL20/IGFBP5/TNFAIP3/EPS8L1/CSNK1G2/IL6ST/APLNR/MAP3K7/SKIL/IL1RN/SULF1/DUSP6/LPAR6/IGFBP4/BCL10/MCL1/IGFBP3/AUTS2/SHC1/MST1R/OTUD4/CD14/ PRNP/PAK2/F2R/SPRY4/HMGB1/SMURF1/EPS8L3/GSK3B/HIF1A/JUP/ LTBP1/ATP2B4/MAP4K4
Regulation of cell communication	1.60E-29	GSK3B/ATP2C1/TRIM38/ECM1/SLC20A1/CANT1/ZDHHC17/CFLAR/RHBDF1/GPRC5A/TRIB2/TRAF4/RIPK2/CAV2/CSNK1A1/CBLB/CCL20/IGFBP5/TNFAIP3/EPS8L1/CSNK1G2/IL6ST/APLNR/ANXA1/MAP3K7/SKIL/IL1RN/SULF1/DUSP6/LPAR6/IGFBP4/BCL10/MCL1/IGFBP3/TCF7L2/AUTS2/SHC1/MST1R/OTUD4/CD14/PRNP/PAK2/F2R/SPRY4/HMGB1/SMURF1/EPS8L3/HIF1A/JUP/LTBP1
Response to external stimulus	1.84E-28	PLXND1/OPN3/SBNO2/BNIP3L/COL1A1/VEGFA/BCL6/PLXNA1/CCL20/PLAU/TNFAIP6/EFNB2/RAC2/PALLD/ANXA1/OASL/RAC1/IL1RN/IFITM3/EPHA2/AUTS2/CXCL5/CD14/ANXA2/IFITM2/IFITM1/HMGB1/PLXNB2/CD47/SERPINE1/APOE/IL6ST/SERPINE2/ADAM17/TFPI/USP33/SSH1/NFKBIA/CAV1/MET/OAS3/GAPDH/STAT1/TNFAIP3/SLPI IRF1/LGALS3/POSTN/PUM1/THBS1/LEF1

**Table 2:** Biological processes in down-regulated genes.

Functional category	P-value cut off [FDR]	Top 50 Genes
Protein targeting to ER	1.15E-14	SEC63/SEC61A1/SGTA/SEC61B/SPCS1/SRP72/SRP54/TRAM1/SSR3/RPL18/RPL31/RPS5/RPL6/RPL3/RPL24/RPL22/RPS10/RPL35/RPS6/RPL11/RPL7/RPL29/RPL9/RPS9/RPS7/RPL15/ RPLP2/RPS17/RPL35A/RPL14/RPL10A/RPS28
Cellular localization	9.96E-11	ITGB3/CSNK2A2/HUWE1/SEC63/KIF1B/SEC61A1/SPAG4/IPO5/PCM1/DLG3/TMED2/ AAAS/TIMM13/SGSM3/SEC23B/PARD6A/SLC6A2/GDAP1/SLC17A7/SGTA/BET1/SEC61B/ DNM1/GOSR2/IFT20/COPZ1/TBC1D30/SPCS1/ARL1/KIF1C/CALY/DLG4/PLCE1/USO1/ PEX5/TEX261/CPLX2/PARD3/PEX10/CLMN/TMED3/RAB26/STXBP6/TMED10/ PLA2G1B/GPHN/TNKS/TOMM20/SRP72/RNF186
Regulation of transport	3.54E-10	ITGB3/CSNK2A2/HUWE1/NEDD4L/PTGER3/CACNA1I/CHGA/HRH3/HOMER2/SCN1B/ POMC/FXYD2/CPLX2/CRH/SHANK1/SHANK2/CACNB2/PLA2G1B/KCNE1/BRSK2/ CD22/FOXP3/KCNQ1/TNK2/KCNAB2/PDGFB/PLTP/AKAP7/PTK2B/EPO/EGF/AZIN1/ NRG1/STXBP6/INSR/PRTN3/HTT/UNC13B/SCAMP5/ICA1/ABCC8/CACNA2D2/NADK/ MAPK8IP2/IPO5/REEP1/FGFR1/CADPS2/GNAO1/GNAS
Cellular protein localization	4.71E-10	CSNK2A2/HUWE1/SEC63/SEC61A1/SPAG4/IPO5/PCM1/DLG3/TMED2/TIMM13/SGSM3/GDAP1/ SGTA/SEC61B/GOSR2/IFT20/COPZ1/TBC1D30/SPCS1/ARL1/DLG4/USO1/PEX5/PEX10/CLMN/ TMED3/RAB26/STXBP6/TMED10/GPHN/TNKS/TOMM20/SRP72/RNF186/NPM1/RAB11B/ RAB40C/CCHCR1/SRP54/CLU/BRSK2/NEDD4L/REEP1/POLDIP3/SAMM50/ UBL4A/GGA2/PEX16/EGF/PML
Cell-cell signaling	1.86E-08	RAB26/TLE2/SCGN/DLG3/NOS1/TYRO3/CHGA/HRH3/SLC6A2/SLC17A7/GRIK5/RNF43/POMC/SLC12A4/DLG4/CHRM3/EGF/CPLX2/CRH/NTRK2/CACNA1B/DVL3/SHANK1/SHANK2/CACNB2/OR10H2/TNKS/WNT7B/UNC13B/GRB10/ICA1/ABCC8/CACNA2D2/NADK/MAPK8IP2/KIF1B/LZTS1/FGFR3/CSNK2A2/NLE1/FGFR1/CADPS2/GNAS/P2RX6/PACSIN2/SYNGR1/HNF4A/SYP/DMPK/ETV2
Cellular catabolic process	3.09E-08	CSNK2A2/NEDD4L/TRHDE/UBE2D4/BCKDHB/HADHA/HUWE1/ANAPC5/NOS1/ CYP2D6/KCTD17/NPRL3/QPRT/SGTA/ZNRF4/RNF43/FBXO9/DAP/NPRL2/ AADAC/ACADL/CLU/PRDX4/SARDH/PSMF1/CYP2E1/HBZ/UBE4B/PKLR/ AMT/RNF144A/EXOG/SPSB3/NEU3/PSMD6/ANPEP/ENDOG/PRDX2/ CTSW/RNF186/SHMT2/SND1/DNASE1/BCKDHA/BRSK2/ HERPUD1/CIRBP/SEC61B/XPNPEP1/
Intracellular transport	7.85E-08	CSNK2A2/HUWE1/SEC63/KIF1B/SEC61A1/IPO5/PCM1/TMED2/AAAS/TIMM13/SGSM3/SEC23B/GDAP1/SGTA/BET1/SEC61B/DNM1/GOSR2/IFT20/COPZ1/TBC1D30/SPCS1/ARL1/KIF1C/CALY/ USO1/PEX5/TEX261/CPLX2/PEX10/CLMN/TMED3/RAB26/TMED10/TOMM20/SRP72/NPM1/ RAB11B/RAB40C/UNC13B/CCHCR1/ARFGAP3/SRP54/CLU/BRSK2/POLDIP3/SAMM50/ CHGA/TRIM27/PHB2
Cellular homeostasis	9.32E-08	TBXA2R/PTGER3/ATP12A/SCGN/SLC4A4/MT3/DMPK/PRDX4/SLC12A4/MT1G/PLCE1/SLC39A8/ATP1A1/PRDX2/PLA2G1B/PTGER4/CMKLR1/PLCB1/ATP4B/HERPUD1/CCKBR/GLP1R/EPO/ CIB2/CACNA1C/HTT/UNC13B/AQP1/CD24/ABCC8/NADK/TXNL1/TYRO3/XBP1/TXN2/ SLC17A7/GRIK5/P2RX1/STC2/PTK2B/ATF4/GATA4/PML/PDIA6/CRH/SYBU/ PTGES2/SIDT2/PTPRN2/TIAM1
Response to external stimulus	2.44E-07	PGLYRP1/BCKDHB/NOS1/SEMA6A/EFNA2/CCL22/NPRL3/CCL24/CXCL12/PPARD/DAP/NPRL2/TNFRSF8/EPX/MTUS1/EPHB2/SEMA4F/CLPS/RHO/CRADD/PLA2G1B/REG1B/CMKLR1/ EPHB3/PDK4/ASNS/FGFR1/XBP1/PDGFB/CHGA/GHSR/FOXA2/IRF5/CIB2/KLK3/ DGKQ/TTN/TRIM44/PTGER4/BNIP3/GRK1/SLIT1/PDGFA/ELANE/NFKBIL1/ AQP1/PDK2/ABCC8/TBXA2R/FOXP3

### Investigation of the protein network existing in the extracellular matrix

**Figure 4:**
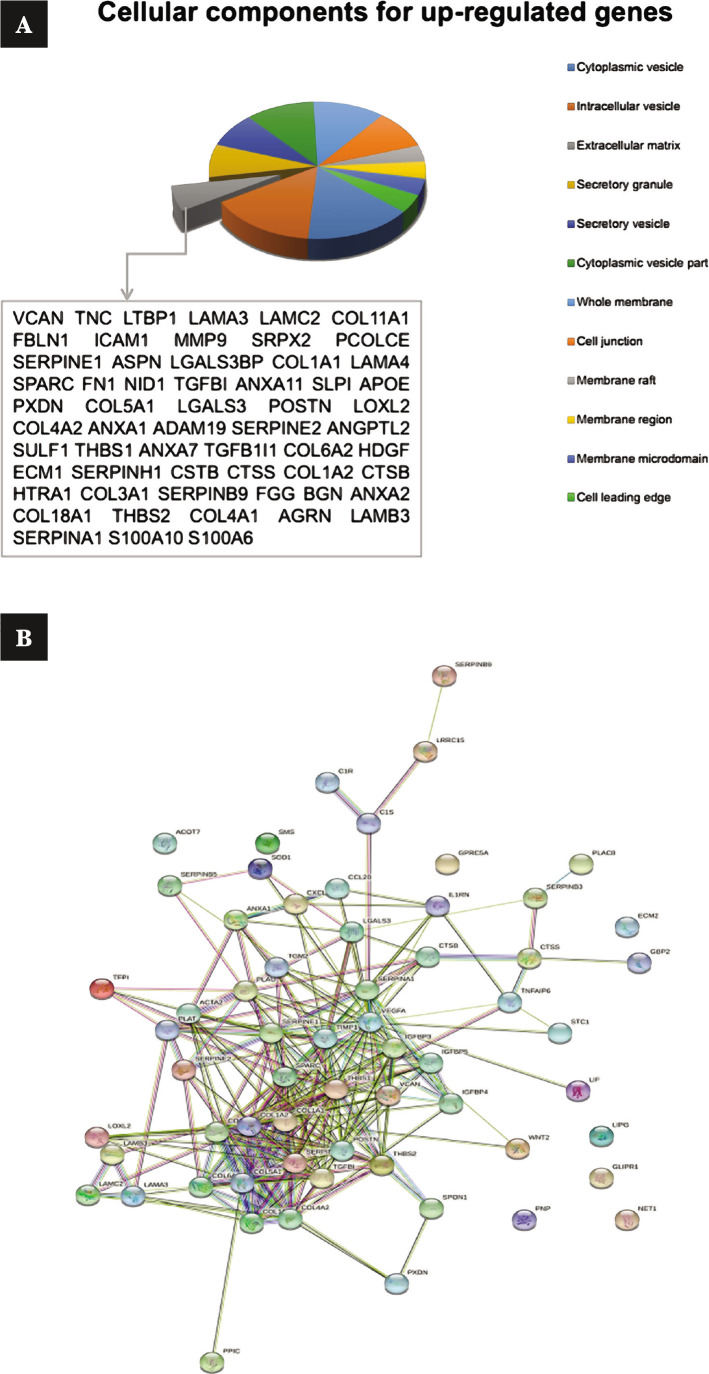
A – protein products in terms of cellular components were measured in up-regulated genes, and genes whose protein product is released into the extracellular matrix were isolated; B – a protein network has been mapped for these genes.

Sixty-two genes of the extracellular matrix were entered into the STRING database for constructing PPI. This network had 62 nodes and 254 edges (Figure 3 A, B). The average node degree of the network was 8.19, and the degree of 23 nodes was more than average. Hub genes were selected using analysis by 12 topological algorithms, and finally, COL1A1, COL1A2, POSTN, COL3A1, SPARC, THBS2, COL11A1, VCAN, SERPINH1, SERPINE1, IGFBP3, ACTA2, PLAU were chosen as hub genes (Figure 4 A, B, Table 3).

**Table 3:** The pathway of studying the topology of proteins in the extracellular matrix use Cytoescape.

Method	Top 30 genes
Maximal Clique Centrality	COL1A1, COL1A2, POSTN, COL3A1, SPARC, COL5A1, THBS2, THBS1, COL11A1, COL4A2, VCAN, TIMP1, COL6A2, SERPINH1, SERPINE1, IGFBP3, VEGFA, IGFBP5, TGFBI, SERPINA1, ACTA2, LOXL2, IGFBP4, PLAU, PLAT, CTSB, TGM2, SERPINE2, LGALS3, IL1RN
Density of Maximum Neighborhood Component	IGFBP5, COL11A1, POSTN, COL1A2, COL3A1, THBS2, COL6A2, VCAN, TGFBI, LOXL2, SPARC, SERPINH1, IGFBP3, COL1A1, COL5A1, SERPINE1, TGM2, ACTA2, COL4A2, SERPINA1, IGFBP4, THBS1, PLAT, CTSB, TIMP1, TFPI, PLAU, LAMC2, CXCL5, SERPINE2
Maximum Neighborhood Component	TIMP1, VEGFA, THBS1, COL1A1, COL1A2, SPARC, POSTN, COL3A1, COL5A1, SERPINE1, COL4A2, THBS2, VCAN, IGFBP3, COL11A1, SERPINH1, IGFBP5, SERPINA1, COL6A2, TGFBI, PLAU, ACTA2, PLAT, IGFBP4, SERPINE2, LGALS3, LOXL2, CTSB, IL1RN, ANXA1
Degree	VEGFA, TIMP1, THBS1, COL1A1, COL1A2, SPARC, COL5A1, POSTN, COL3A1, SERPINE1, COL4A2, THBS2, VCAN, IGFBP3, SERPINA1, COL11A1, SERPINH1, IGFBP5, COL6A2, TGFBI, PLAU, ACTA2, PLAT, LGALS3, IGFBP4, CTSB, SERPINE2, ANXA1, LOXL2, TGM2
Edge Percolated Component	TIMP1, COL1A1, THBS1, SPARC, VEGFA, COL3A1, COL1A2, POSTN, SERPINE1, IGFBP3, COL5A1, THBS2, VCAN, COL4A2, COL11A1, IGFBP5, SERPINH1, TGFBI, COL6A2, SERPINA1, PLAU, ACTA2, IGFBP4, PLAT, SERPINE2, LOXL2, TGM2, LGALS3, CTSB, ANXA1
Bottleneck	VEGFA, COL1A1, THBS1, SERPINA1, COL5A1, LGALS3, TNFAIP6, C1S, PLAT, SPARC, THBS2, VCAN, TIMP1, SERPINH1, IGFBP3, PLAU, CTSS, SPON1, SERPINB3, LRRC15, COL1A2, POSTN, COL3A1, COL11A1, COL4A2, COL6A2, SERPINE1, IGFBP5, TGFBI, ACTA2
EcCentricity	SPARC, THBS1, VCAN, TIMP1, SERPINE1, IGFBP3, VEGFA, IGFBP5, SERPINA1, IGFBP4, PLAT, CTSB, TGM2, IL1RN, COL1A1, COL1A2, POSTN, COL3A1, COL5A1, THBS2, COL11A1, COL4A2, COL6A2, SERPINH1, TGFBI, ACTA2, PLAU, SERPINE2, LGALS3, ANXA1
Closeness	VEGFA, TIMP1, THBS1, COL1A1, SPARC, COL1A2, SERPINE1, POSTN, COL5A1, COL3A1, VCAN, THBS2, IGFBP3, SERPINA1, COL4A2, IGFBP5, SERPINH1, COL11A1, TGFBI, PLAU, PLAT, COL6A2, ACTA2, LGALS3, CTSB, TGM2, IGFBP4, ANXA1, IL1RN, SERPINE2
Radiality	TIMP1, VEGFA, THBS1, SPARC, COL1A1, SERPINE1, COL1A2, VCAN, SERPINA1, POSTN, IGFBP3, COL5A1, THBS2, COL3A1, IGFBP5, SERPINH1, COL4A2, PLAU, PLAT, TGFBI, COL11A1, CTSB, LGALS3, ACTA2, TGM2, IGFBP4, IL1RN, TNFAIP6, COL6A2, ANXA1
Betweenness	VEGFA, SERPINA1, THBS1, TIMP1, C1S, COL5A1, LGALS3, CTSB, CTSS, COL1A1, SERPINB3, LRRC15, SERPINE1, TNFAIP6, SPARC, COL4A2, SERPINH1, VCAN, PLAT, THBS2, COL1A2, PLAU, POSTN, IGFBP3, COL3A1, ACTA2, ANXA1, IL1RN, COL11A1, LAMA3
Stress	VEGFA, THBS1, SERPINA1, TIMP1, C1S, COL5A1, COL1A1, SERPINE1, LGALS3, SPARC, CTSB, COL1A2, CTSS, POSTN, COL4A2, VCAN, SERPINH1, THBS2, TNFAIP6, COL3A1, LRRC15, SERPINB3, IGFBP3, PLAT, PLAU, IGFBP5, COL11A1, TGFBI, ACTA2, SERPINE2
Clustering Coefficient	LOXL2, WNT2, STC1, TFPI, IGFBP5, COL11A1, COL6A2, TGFBI, IGFBP4, THBS2, LAMC2, CXCL5, SERPINH1, POSTN, COL3A1, VCAN, ACTA2, SOD1, LAMB3, SERPINB5, COL1A2, IGFBP3, SPARC, TGM2, IL1RN, SERPINE1, COL1A1, CTSB, SERPINE2, PLAU
Intersected genes	COL1A1, COL1A2, POSTN, COL3A1, SPARC, THBS2, COL11A1, VCAN, SERPINH1, SERPINE1, IGFBP3, ACTA2, PLAU

### Verification of key genes in pancreatic cancer samples through the Cancer Genome Atlas

**Figure 5:**
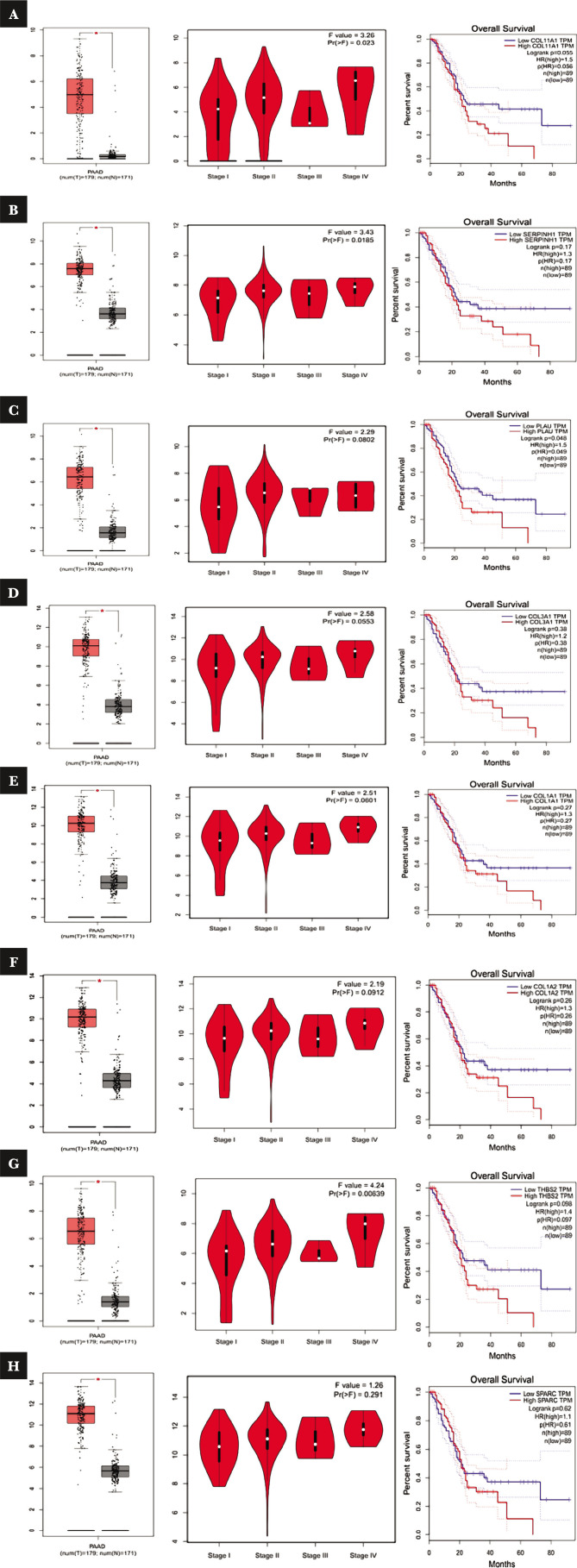
Using the GEPIA and TCGA databases and evaluating the topology of selected proteins shown in Table 2, we isolated eight significant genes that may play a potential role in the non-invasive to invasive stages of pancreatic cancer. Gene expression was compared in human samples with pancreatic cancer and healthy individuals. Stage plots and survival plots were diagramed separately for each protein. A – COL11A1; B – SerpinH1; C – PLAU; D – COL3A1; E – COL1A1 F – COL1A2; G – THBS2; H – SPARC.

Thirteen hub genes COL1A1, COL1A2, POSTN, COL3A1, SPARC, THBS2, COL11A1, VCAN, SERPINH1, SERPINE1, IGFBP3, ACTA2, and PLAU were investigated in TCGA to verify their roles in PAAD cancer. These genes were surveyed to demonstrate expression differences between normal and PAAD cancer samples, and box plots were drawn. As shown in Figure 5, all 13 genes demonstrated statistically significant expression differences. Furthermore, survival analysis of these genes was conducted with the Kaplan-Meier method, and as a result, eight genes SPARC, THBS2, COL11A1, COL1A1, COL1A2, COL3A1, SERPINH1, and PLAU were obtained with the highest mortality rate for PAAD patients. In other words, patients with a high expression level of these genes showed less survival. We showed expression levels of the genes in various stages of the disease using Violin plots drawn in TCGA. Although all eight genes had expressed in all stages, COLL11A1 demonstrated the highest expression difference between stages I and IV. Finally, THBS2 and COL3A1 could be mentioned, respectively. In general, expression of all genes except PLAU increased from beginning to stage II continuously but decreased in stage III and then rose in stage IV again. However, the increasing trend of PLAU continued until stage III and fell in stage IV.

## DISCUSSION

The selection of biomarkers from the tissues of cancer patients is essential in discussing early detection and diagnosis. Although the prognosis for pancreatic cancer is deficient in developing countries, due to the numerous economic and cultural problems they face, the lack of public awareness and the lack of annual routine tests to check individuals' health, treatment management, and diagnosis make pancreatic cancer very severe. As a result, it increases the mortality rate of patients [21, 22]. Therefore, in the present study, we aimed to select important biomarkers for pancreatic cancer using continuous bioinformatics analysis and use it as a diagnostic platform for candidates in the community.

In the first part, we isolated the signaling pathways involved in non-invasive to invasive stages of pancreatic cancer. Based on this analysis, cell adhesion was selected as one of the critical pathways in this study. There are several genes involved in cell adhesion that are important for both healthy cells and cancer cells. FAK is one of these essential genes, which was significantly higher in patients with pancreatic cancer than in healthy individuals, which controlled the expression of FAK through small molecules that effectively reduced the invasion of pancreatic cancer cells [23]. Also, in terms of the microenvironment of pancreatic cancer cells, we can mention the high density of various organs in the body and also the high diversity of cells, including immune cells and blood cells, and cell adhesion, which play a very important role in organizing tissues and regular activity of this volume of cells. In pancreatic cancer, the disorder can affect other parts of the body [24]. Another pathway along cell adhesion that plays a key role in cancer cells' survival is the extracellular matrix. In the study by Begum et al., a clear relationship was shown between cell adhesion and the extracellular matrix. This relationship showed that extracellular matrix and cell adhesion are two inseparable components that are very important for the survival and invasion of pancreatic cancer cells to other internal body organs. High FAK expression is one of the crucial factors in initiating pancreatic cancer invasion [25]. The continuation of the present study, based on the analysis of genes and protein products involved in the extracellular matrix using Cytoscape software, examined the gene topology, which finally identified eight genes as candidate genes essential for further study of biomarkers for pancreatic cancer.

Cancer cells convert surrounding fibroblasts to activated cancer-associated fibroblasts (activated CAFs), which secrete some products such as collagens, growth factors, and enzymes that promote angiogenesis, invasion, and metastasis in tumors [26]. COL11A1 is a protein-coding gene that encodes one of the two alpha chains of type XI collagen [27]. Chondrocytes, mesenchymal stem cells, and osteoblasts express COL11A1, but normal epithelial cells and quiescent fibroblasts of different body locations do not express this gene and the derived products [27]. High-level expression of COL11A1 is related to mesenchyme-derived tumors, cancer-associated stromal cells, and carcinoma progression. This gene is not expressed under standard conditions in the pancreas and benign pathological processes such as pancreatitis, so it could be a remarkable biomarker for PAAD cancer [27]. As reported in different studies, pancreatic cancer cells expressed a high level of COL11A1, and this high expression has a good value as a biomarker of activated CAFs and PAAD cancer [20, 26, 28, 29]. Sun et al. demonstrated that the high expression level of COLL11A1 in pancreatic cancer patients was related to poor prognosis and high mortal rate of PAAD patients based on an immunohistochemical assay [30]. In the present study, the difference in COL11A1 expression between normal and PAAD samples was so evident that the normal samples showed almost zero expression. The high expression level of COL11A1 is associated with patients' high mortality rate, as illustrated in Figure 5 A. The five-year- survival rate is 10%, and after about 70 months, the survival rate reaches 0%. Also, the stage plot analysis of COL11A1 showed that the gene expression level except for the stage 3 continuously increases. Therefore, this gene could be an excellent suggestion for biomarkers, which is not expressed in healthy people but expressed in PAAD patients increasingly during the disease's progression. As the expression rate goes up, the survival rate goes down.

Serpin peptidase inhibitor clade H member1 (SerpinH1) is an essential chaperone for the correct folding and secretion of different collagen types [31]. Kurahara et al. reported that HMP19 suppresses PAAD tumor growth and metastasis. HMP19 interacts with some signaling proteins, such as SerpinH1, which is detected in PAAD invasion and progression [32]. Different studies presented that in numerous cancers, including glioma, pancreatic cancer, cervical and lung cancer, the expression level of SERPINH1 rises [31, 33, 34]. Cao et al. showed that the expression level of SERPINH1 in gastric cancer plasma samples is significantly higher than in normal plasma samples [31]. Moreover, a high level of SERPINH1 is reported in clear cell renal cell carcinoma [33].

Normal and PAAD samples had significantly different levels of SERPINH1 expression in the present study. Subsequently, Figure 5 B showed that the high expression level of SERPINH1 leads to a low survival rate in PAAD patients, so that during the 75 month, the mortality rate was proximately 100%, and five year- survival rate was 20%. The expression level's differences in various stages are not very much, but the expression in stage 4 is more than in stage 1. Consequently, SERPINH1 is another suggested biomarker significantly overexpressed in PAAD patients, decreasing the survival rate during progression.

Other studies have identified PLAU and COL3A1 as hub genes [35–37]. PLAU is a protein-coding gene on chromosome 10 that encodes urokinase-type plasminogen activator (uPA) with serine protease activity. This 53 kDa protein is secreted to the extracellular matrix and converts plasminogen to plasmin [38–40]. Studies showed overexpression of uPA in cancer cells. uPA binds to the uPA receptor and activates a proteolytic cascade that degrades the extracellular components [40]. uPA, in interaction with transcription factors, regulates the expression of genes related to stem-like characterization in pancreatic cancer cells [41]. Up-regulation of uPA during gemcitabine-induced endoplasmic reticular stress inhibits mitochondrial apoptosis and leads to chemotherapy resistance in pancreatic cancer [42]. Xu et al. reported the inhibitory effect of Triptolide on endothelial-to-mesenchymal transition via targeting PLAU [43]. The boxplot for PLAU showed that expression of this gene was significantly increased in PAAD patients (Figure 5 C). The Violin plot indicated a pronounced increase of expression between stages II and I; however, gene expression was poorly changed in stages III and IV. The high PLAU group had decreased overall survival. Percent survival for high PLAU patients was under 0.2 after 60 months, while it was almost 0.4 for low PLAU patients.

COL3A1 is located on chromosome 2 and encodes the alpha 1 chain of type 3 collagen. COL3A1 has high expression levels in the gallbladder, placenta, urinary bladder, and endometrium [44]. Studies reported up-regulated levels of COL3A1 by cancer progression. Huo et al. identified COL3A1 knockdown disturbed migration and proliferation of cancer cells in glioma [45]. Yu Shi et al. reported down-regulation of methyl transferase-like three increased expression of COL3A1 as its target and elevated migration and invasion of cancer cells in breast cancer [46]. Desmoplasia has a crucial role in drug resistance and the progression of pancreatic cancer, and a study identified that a combination of gemcitabine and EC359 affected desmoplasia by down-regulating COL3A1 [47]. Our analyses showed that COL3A1 had significantly increased levels of expression in PAAD patients. Expression of COL3A1 increased in stage II while it fell in stage III and increased in stage IV. The survival plot showed that low COL3A1 patients had higher overall survival than high COL3A1 patients. The survival rate percentage was the same for low and high COL3A1 after 20 months. Low COL3A1 patients had a slight decrease in percent survival for 40-month overall survival. After 40 months, the survival rate percentage was almost constant; however, it was reduced in high COL3A1 after 20 months. This reduced overall survival demonstrates the importance of COL3A1 expression in pancreatic cancer prognosis (Figure 5 D).

COL1A1 gene expresses mRNA, which is translated into the pro-alpha1 chain of COL1A1, forming a fibrillary form collage type 1 collagen. COL1A1 is more expressed in the gall bladder and urinary bladder. The role of this gene in metastasis and invasion in various cancers has been studied. Liu et al. reported that CXCR4-mediated knockdown of COL1A1 reduced the invasive potency in breast cancer [48]. In another study, Zhang et al. stated that COL1A1 increased metastasis in colorectal cancer by regulating critical genes in the WNT/PCP signaling pathway, which are involved in cell morphology, metastasis, and adhesion [49]. Type 1 collagen enhances epithelial-to-mesenchymal transition by targeting β1-integrin aNd, followed by GL1 up-regulation. Palmatine suppresses the growth of cancer cells via targeting COL1A1 in a GL1-dependent manner [49]. As demonstrated in Figure 5 E, the COL1A1 gene had a significantly higher expression in pancreatic cancer cells. Expression of COL1A1 enhanced in stages II and III; however, it showed a slight decrease in phase III. The percent survival rate of low COL1A1 patients was reduced to 0.4 until the 20^th^ month, while overall survival of high COL1A1 had a specific reduction after the 20^th^ month, and the percent survival rate was under 0.2 in the 60^th^ month.

In this study, Figure 5 F indicates normal and PAAD samples had significantly different levels of COL1A2 expression. The high expression level of COL1A2 is associated with a high mortality rate of patients, as has been confirmed, in which the five-year- survival rate is almost 15%. After about 75 months, the survival rate reaches 0%. Also, stage plot analysis of COL1A2 showed that the gene's expression level, except for stage 3 and stage 4, continuously increases.

In addition, some researchers have reported that COL1A2 may be directly involved in pancreatic cancer proliferation, migration, and progression of the invasion [50].

Thrombospondin-2 (THBS2) is a disulfide-linked homotrimeric glycoprotein, and its function is to facilitate interactions between cells to the matrix. Human precursor pancreatic intraepithelial neoplasias (PanIN) organoids secrete or release THBS2. PanIN is detectable in PDAC patients up to 10 years before the advanced stage of PDAC. Because of this, early detection with THBS2 as a biomarker is possible [51, 52]. A study on mouse models shows that THBS2 probably is a host anti-tumor defense mechanism with an anti-angiogenic mechanism. Naturally, the expression of THBS2 in a patient with cancer increases [53]. The high level of THBS2 in the plasma of PDAC patients has been demonstrated, and resectable PDAC stage I cancer and advanced stage III/IV can differ with the concentration of THBS2 in plasma [52]. As shown in Figure 5 G, the difference in THBS2 expression between normal and PAAD samples had an obvious different level of THBS2 expression in the present study. The high expression level of THBS2 is associated with a high mortality rate of patients, of which a five-year survival rate is 10%. After about 70 months, the survival rate reaches 0%. Stage plot analysis of THBS2 confirms that the expression level of the gene except for stage 3 and stage 4 continuously increases, so this gene has the potential to become a biomarker.

SPARC, also known as osteonectin, is a matricellular protein involved in various cellular processes such as proliferation, apoptosis, adhesion, migration, matrix remodeling, and angiogenesis [54]. SPARC was expressed in low and normal levels in adult tissues with high ECM turnovers, such as bone and the gut epithelium, but in PDAC patients, a high SPARC level has been observed [55, 56]. According to studies, SPARC is concentrated in the tumor stroma and overexpressed in breast cancer, lung cancer, melanoma cancer, and gastric tumors [57, 58]. Recent bioinformatics analysis on a prognostic biomarker in pancreatic ductal adenocarcinoma suggests SPARC as a potential biomarker for this disease [53, 59]. As shown in Figure 5 H, normal and PAAD samples had a different recognizable SPARC expression level in this study. The high expression level of SPARC leads to the low survival rate of PAAD patients, so that during 70 months, the mortality rate was proximately 100%, and the five-year survival rate was 10% which is not very much based on the differences in the expression level in various stages.

## CONCLUSION

In general, selecting a panel of appropriate biomarkers present in the extracellular matrix and extracted through various human flows is the right solution for early detection, followed by proper treatment management and mortality reduction. This study focuses on SPARC, THBS2, COL11A1, COL1A1, COL1A2, COL3A1, SERPINH1, and PLAU proteins released into the extracellular matrix that provided various biomarkers for non-invasive to invasive stages of pancreatic cancer, which require more extensive studies to investigate and find more precise mechanisms of these proteins.

## Data Availability

All data generated or analyzed during this study are included in this published article.

## Personal thanks

We thank all colleagues in the core facilities for their support.
